# SEXUAL AND GENDER MINORITY EXPERIENCES AND PREFERENCES WITH LONG-TERM SERVICES AND SUPPORT PROVIDERS

**DOI:** 10.1093/geroni/igae098.1614

**Published:** 2024-12-31

**Authors:** Rajean Moone

**Affiliations:** University of Minnesota, St. Paul, Minnesota, United States

## Abstract

Sexual and gender minority (SGM) older adults are at higher risk for complex, health related conditions as they age. Coupled with smaller social support networks they often must rely on formal long term services and support (LTSS) providers. However, due to a history of personal and systemic discrimination many SGM older adults limit access to LTSS. To gain further insight into SGM older adult experiences, a survey needs assessment consisting of 46 self-reported questions was conducted in Minnesota in 2022, replicating similar waves in 2002 and 2012. 354 respondents met the inclusion criteria. Half of the respondents (50% of sexual minority and 53% of gender minority) reported personally experiencing harassment, abuse, or violence and 75% reported knowing someone who had. At the same time, respondents overwhelmingly expressed desire for integrated services that are SGM welcoming rather than SGM exclusive. A majority (57%) had some confidence in being treated with dignity and respect by health care professionals at the end of life. Respondents also indicated they would be more inclined to utilize LTSS where staff participated in SGM training (92%). Results show need for LTSS provider education on SGM trauma-informed and age-inclusivity and targeting of SMG older adults aging alone.

